# Framing the crisis: X/Twitter discourse on Ukrainian war refugees in Poland

**DOI:** 10.1371/journal.pone.0346666

**Published:** 2026-05-05

**Authors:** Tomasz Piróg, Rafał Olszowski, Piotr Pięta, Tomasz Masłyk

**Affiliations:** 1 Faculty of Humanities, AGH University of Krakow, Poland; 2 Faculty of Electrical Engineering, Automatics, Computer Science and Biomedical Engineering, AGH University of Krakow, Poland; Polish Academy of Sciences: Polska Akademia Nauk, POLAND

## Abstract

Social media play a crucial role in shaping public perceptions of migration during crises. This study examines how discourse on Ukrainians in Poland was framed on X/Twitter during the first year of Russia’s full-scale invasion of Ukraine (2022), contributing to research on framing and crisis communication in networked social media environments. Triangulating both quantitative and qualitative methods, we analysed 55,035 Polish-language posts containing the keywords “Ukrainians” and “in Poland.” The analysis combined frame analysis with several complementary methods: stance detection, network analysis, and engagement measurement based on a repost conversion rate. Our findings indicate that while posts displaying negative attitudes dominated in quantity (60%), positive posts had the highest conversion rate. The dominant frames included conflict, international relations and assistance to Ukrainians, highlighting a notable polarization of discourse. Network analysis identified four key user groups with distinct political affiliations. Right-wing government supporters (G1) and the liberal opposition (G4) expressed pro-Ukrainian sentiments, while users with nationalist views (G2) were critical of the influx of Ukrainian migrants. Although Group G2 generated the highest number of posts, their reach was limited due to lower engagement. In contrast, a smaller, politically diverse group (G3) maintained a more neutral stance. These findings demonstrate how integrating frame analysis with network analysis helps capture the relationship between interpretative frames and the structural dynamics of online communication. While solidarity with Ukrainians was strong in 2022, future research should examine how long-term migration processes transform online discourse over time.

## 1. Introduction

Armed conflicts and large-scale migratory movements represent not only humanitarian crises but also pivotal moments of intensified public communication. Social media platforms, in particular, serve as arenas where perceptions of crises are rapidly shaped and contested. Against this backdrop, our article examines the Polish-language discourse on Ukrainians on X/Twitter between March and December 2022 (formerly Twitter; to ensure clarity, the term X/Twitter is used throughout this text). The analysis focuses on user reactions to the crisis triggered by the massive influx of war refugees from a neighbouring country following Russia’s invasion of Ukraine. The study contributes to the growing research field known as *crisis informatics* (Hagar, 2007), which investigates how new media are used in situations of conflict and emergency. As Palen et al. (2009: 467–480) emphasize, this perspective views emergency response as an expanded social system where information circulates between official and public actors. Within this framework, the present study centres on the X/Twitter platform, which often surpasses traditional media by providing immediate access to news and debates. Its value in crisis situations stems not only from its networked structure but also from the presence of influential opinion leaders: politicians, journalists, social activists, entrepreneurs, whose posts spark reactions that extend their reach across broader audiences.

The analysis covers the most frequently circulated posts from the first year of the war, selected according to the keywords “Ukrainians” and “in Poland”. The aim of the study is to deepen our understanding of how opinions about refugees were shaped in the most popular posts published at the beginning of the refugee crisis. It examines the characteristics that define posts and their authors, as well as the features that distinguish them and reflect polarisation. Which frames and stances are employed in the posts, and how are the authors positioned within user network clusters? Answering these questions provides crucial insights into the early stages of war, when public opinion is shaped by rapidly spreading narratives, and the entire process is not yet supported by comprehensive social research. This underscores the significance of social media analysis, both as a vehicle for disinformation and as a platform for disseminating news and public emotions in response to unfolding events.

Simultaneously, the article addresses the question of how the triangulation of social network clustering and stance detection contributes to frame analysis in social media. It argues that the explanatory power of frame analysis can be enhanced through a mixed approach that moves beyond the existing debate on the advantages and disadvantages of qualitative and quantitative methods in framing research [1]. Our triangulation mitigates the limitations of small, manually coded samples typical of qualitative studies and simultaneously enhances the interpretive precision often lacking in fully automated analyses. It therefore provides a balanced framework for studying online discourse during crises, where speed, accuracy, and contextual sensitivity are equally important. In addition, it combines two different frame classifications (generic and issue-specific frames) with stance analysis, which deepens our understanding of how frames are used.

### 1.1. Brief background of the study

The history of Polish – Ukrainian relations is complex, marked by centuries of coexistence within shared political frameworks as well as by episodes of violent rupture. The two nations are linked by linguistic and cultural affinities that evolved in a common civilizational space, yet their collective memories remain burdened by the traumas of the twentieth century, including the Volhynian massacres and post-war forced resettlements. After the fall of communism and the collapse of the Soviet Union, successive Polish governments supported the pro-Western orientation within Ukrainian politics, recognizing that integration into European and transatlantic institutions would promote regional stability and serve as a vital component of the national security of both Ukraine and Poland in the face of the enduring threat posed by Russia.

In the initial phase of the 2022 Russo-Ukrainian conflict, the refugee crisis in Poland was accompanied by a significant mobilisation of civil society, which demonstrated profound solidarity with migrants and, in some areas, acted ahead of state initiatives [2]. The Polish government introduced a system of subsidies for individuals and businesses providing temporary accommodation to Ukrainians, thereby eliminating the need to establish refugee centres. Legislative reforms granted Ukrainian refugees access to healthcare, the education system, social benefits, the labour market, and the right to establish businesses. Notably, similar privileges were not extended to refugees from other countries, such as those entering Poland from Belarus [3,4].

One year prior to Russia’s invasion of Ukraine, there were 307,700 Ukrainians in Poland holding valid residence permits [5]. The outbreak of the Russo-Ukrainian War in 2022 marked a decisive turning point, reshaping demographic trends and triggering an unprecedented social and communicative response to the arrival of war refugees. As a result of the conflict, over one million Ukrainian refugees, predominantly women and children, sought shelter in Poland [6,7]. Notably, the number of non-Ukrainian foreigners residing in Poland also increased substantially between 2014 and 2024, rising from 134,100–399,600 [5]. Despite these demographic shifts, the country is almost mono-ethnical (in 2002, 96.7% of the Polish population identified themselves as Polish; in 2021, 89.2%) and its population declines, primarily due to emigration and adverse demographic trends [8]. These characteristics of Polish society constitute the circumstances in which the study was conducted (see S1 Text for details).

## 3. State of research

X/Twitter platform is of particular interest to researchers examining the consequences of migration and refugee crises, encompassing a wide range of studies. These studies include analyses of hate speech, sentiment, and user attitudes towards migrants [9–20], as well as examinations of xenophobic discourse disseminated by political parties and social groups [21–25]. Researchers have also investigated communications from public immigration services [26], non-governmental organizations [27] and hybrid disinformation practices [28–30]. However, the existing academic literature includes only a limited number of advanced studies analyzing the reception of Ukrainian migrants among users of the X/Twitter platform following Russia's full-scale invasion of Ukraine [16].

Research in the field of crisis informatics indicates that in crisis situations, most social media activities are aimed at informing other citizens [31,32]. In the context of emergencies, social media provide extensive opportunities for civic engagement [33]. While a participatory media structure is not the sole condition for societal transformation, social media can nonetheless assume a pivotal function in the organization of political conflicts and protests [34]. The power of new media in this context was first recognised during the Arab Spring, which prompted a large number of publications on the importance of Facebook, X/Twitter and other social media in mobilizing protests and shaping public opinion during political crises [35–38]. During crises social media users tend to respond in a predominantly rational manner rather than with panic. Coping strategies are manifested through practices such as citizen-led reporting, community-oriented computing, and distributed forms of problem-solving [39,40]. Organizations that feel responsible for resolving the crisis (governmental, non-governmental) inform the public and monitor content generated by citizens, while trying to take into account the risks associated with the presence of inaccurate and outdated content on social networks, the uneven distribution of useful information [31,32].

Concurrently, numerous studies have highlighted that the reception of a large number of refugees by a host country can lead to social unrest [41–43]. As a consequence, refugees may experience discrimination, which can adversely affect their mental health [44]. Analyzing communication on X/Twitter is one method for diagnosing the scale of this phenomenon in the online sphere [45]. Some studies suggest that resentment towards refugees on X/Twitter is lower than towards regular migrants [46]. However, a growing body of research indicates that the nature of online discourse regarding refugees evolves as a refugee crisis unfolds. In the initial phase, criticism of refugees is primarily expressed through frustration and opposition to their reception. Over time, this discourse may escalate into open hate speech and discrimination [47]. Anti-immigrant sentiment among X/Twitter users tends to rise when immigration-related topics receive increased coverage in traditional mass media, often driven by refugee crises [17]. Moreover, in geographic areas where individuals from different ethnic and cultural backgrounds interact more frequently, anti-immigrant content on X/Twitter appears less frequently and fails to gain traction [17]). This suggests that the primary driver of antagonistic attitudes is a perceived threat from migrants [48], whereas frequent intergroup contact serves as a mitigating factor [49].

Building on the above theses, we assumed that during the initial phase of refugee crisis, posts from network clusters affiliated with the government or with prior experience in governance would demonstrate greater solidarity with refugees and attract higher popularity (as measured by reposts) than those from clusters representing groups without experience in state responsibility, which sought to build political capital by fuelling conflict and social divisions during a time of crisis.

Our study analyses posts using frame analysis within a real-time crisis window, which is an established research practice [50]. The concept of framing originated in cognitive psychology and was initially used to examine how individuals interpret the world by emphasising specific aspects of reality [51]. Media messages are conveyed to the public through particular frames, which shape how these issues are understood and perceived by audiences [52,53]. While the concept of framing has been widely adopted across the social sciences and humanities [54], its limitations have sparked ongoing debates about methodological precision and the risk of misapplication, especially within media effects research [55,56]. Despite these concerns, the concept has gained influence beyond this specific field of research [57], fostering the integration of various interdisciplinary perspectives. These perspectives focus on how particular phenomena are represented in the media rather than on the effects of such representations [1]. Notable examples include framing analyses of armed conflicts and war [1,58–62], representations of immigration in election campaigns [63], natural disasters [64,65], health risks and healthcare systems [66,67].

In this study, we draw upon the generic frames developed by Semetko and Valkenburg [68]. In order to enhance the accumulation of knowledge and the comparability of findings, they proposed five types of frames: *conflict*, *human interest*, *responsibility*, *economic*, and *morality* frames. These frames constitute universal patterns of media discourse that have the potential to transcend thematic boundaries and are deeply embedded in journalistic routines, such as conflict-seeking and the personalisation of news stories. In addition, we employ issue-specific frames, which were inductively derived from the research material during the pre-analysis stage. This approach has been widely adopted in studies of media content [50]. It narrows the analytical focus to a specific research context, enabling a deeper understanding of it, while simultaneously limiting the comparability of results. We identified five issue-specific frames — *migration flows, migrant features, helping Ukrainians, the Second World War,* and *international relations*. A detailed description of both the generic and issue-specific frames used in this study is provided in the methodology section.

Semetko and Valkenburg’s framing typology has been widely used to analyse how refugee crises are represented in both traditional and social media. Previous research has shown that traditional media most often describe such crises using two of the five generic frames: the *human interest* and *responsibility* frames. This pattern was found in coverage of both the Syrian and Ukrainian refugee crises. The Syrian crisis also generated a substantial amount of content classified under the *conflict* frame, which may be explained by the greater cultural distance between non-European migrants and host societies [69]. Similar findings have been reported in other studies. These indicate, for example, the dominance of the *conflict* frame, particularly in the context of tensions between groups of different religions [70,71], and that more than half of the media content related to irregular migration employs the human interest frame [72]. A clear polarisation of narratives is also evident, linked to the use of opposing generic frames: the *conflict* frame, which highlights threat, and the *human interest* frame, which emphasises solidarity with victims. This polarisation reflects the division between conservative and liberal media outlets [73].

There is evidence to suggest that the distribution of framing frequencies in social media differs from that observed in traditional media, although this topic requires further exploration. Preliminary studies indicate that the *responsibility* and *economic* frames are most frequently used to describe refugee crises [74]. These frames are often associated with negative posts focusing on institutional criticism and on economic anxieties within society related to the influx of refugees. The lower frequency of the *human interest* frame in social media may be linked to the declining role of traditional newsrooms, which previously tended to mitigate crisis coverage by incorporating a more empathetic or human-centred perspective [55].

Based on the aforementioned studies, before conducting our analysis we assumed that, when applying the generic frames, the majority of posts would be classified under the *responsibility* and *conflict* frames. For similar reasons, we also hypothesised that the results concerning issue-specific frames would be quantitatively dominated by posts representing the *helping Ukrainians* and *migrant features* frames. These categories are associated with the actions of institutional actors involved in managing the crisis, as well as with characteristics of refugees that evoke public anxieties and hopes related to the dynamics of social relations.

In our method, we apply stance detection, also known as opinion mining [75,76], to the analysed posts, which allows us to calculate the mean stance value for each frame. We assume that a negative stance reduces reposts, as shown in previous research [77]. Like frame analysis, stance detection is widely used in social media studies, both automatically and manually [78], and has been combined with frame analysis in earlier work [79,80]. Automated analyses allow for the examination of larger samples of posts but tend to be less reliable in qualitative terms. Manual analyses, by contrast, are based on smaller samples, which may limit their validity. Therefore, we used a purposive sample, the selection of which is described in the next section. At the outset of the study, we did not assume which stance would be most prevalent in the posts included in the sample. The only a priori assumption was that social media dynamics promote polarisation. This was expected to be reflected both in the frequency with which frames are used by authors from particular network clusters and in the average stance associated with a given frame.

We additionally apply network analysis, enabled by metadata. This represents an innovative approach within framing research that, to our knowledge, has not yet been implemented, despite the widespread use of other forms of triangulation and the common application of network analysis in studies of social media [81–87]. Network analysis enables us to describe the relationships between users by mapping the structure and characteristics of the network. This allows for the division of the social network into logical clusters (e.g., supporters versus opponents). We employ this division in the content analysis of posts using frame analysis and stance detection, assuming that the results of the network analysis provide information about clusters in which users frame the refugee crisis differently. In addition, using the same corpus of posts, we examine how the crisis is framed and the relative popularity (in terms of engagement or conversions) of these frames. This part of the study draws inspiration from frame contest analysis in social movement research [88]. which serves as a loose conceptual reference for our exploration of social media networks. Our assumption that cluster-level differences will emerge is derived from the well-established finding concerning the presence of competing frames in party-motivated reasoning [89]. We therefore expect that our triangulated approach will enable us to verify how social divisions manifest themselves in the framing of the refugee crisis.

## 4. Research method

Our triangulation combines qualitative frame and stance identification with quantitative indicators of discourse dynamics including network clustering and engagement metrics, based on repost counts. Such integration enables us to link the interpretative layer of frame analysis (what meanings are expressed) with the structural and behavioural layer of communication (how these meanings circulate and gain visibility).

Extraction of unstructured data from X/Twitter has been performed using R scripts through the Application Programming Interface (API) v2 for Academic Research, which enabled researchers to retrieve posts from the entire X/Twitter archive. At the time the data was collected, access to the Twitter API for Academic Research was still possible but was restricted after the company changed its policy in February 2023. The post selection criteria were (i) posts published in the Polish language, (ii) posts containing the keywords “Ukraińcy” (“Ukrainians”), “w Polsce” (“in Poland”), and (iii) posts that were published between 24 February 2022 (12:00 a.m. CET) and 31 December 2022 (11:59 p.m. CET). The time frame selected for this study is related to the date when the Russian Federation invaded Ukraine and the closing date of the first calendar year of the conflict. The X/Twitter users included in the data analysis were those who sent posts with the above-mentioned characteristics during the pre-defined period. Unverified users were also included, as one of the objectives of the study was to analyse message dissemination.

A total of 55,035 posts (original content), reposts (forwarded content), and replies (discussions among users) were collected. These were then extracted, and imported into NodeXL software, which is a professional tool for analysing social media, used in many research projects [90–93]. Then, the data was analysed using the social network analysis approach, as described in the next section. This allowed a network graph to be created. The dataset contained data from 22,470 X/Twitter users (including users who were replied to or mentioned in posts). NodeXL was used to generate network metrics, such as betweenness centrality and network clusters, with a validated methodology used in previous research [94–96]. To produce a visualisation we used the Harel-Koren Multiscale algorithm, a force-directed layout algorithm used for visualising large-scale networks [97]. This algorithm helps to reveal patterns and detect clusters by arranging network nodes in a way that reflects their relationships and proximity. The size of the nodes in the graph were ranked by their betweenness centrality score (BCS) [98]. The BCS measures the influence of a vertex over the flow of information between all other vertices – under the assumption that information flows over the shortest paths among them. The graph’s vertices were grouped by cluster using the Clauset–Newman–Moore (CNM) algorithm [99]. The CNM algorithm operates by optimising modularity, a measure that evaluates the density of connections within communities relative to the density of connections between communities. The algorithm seeks to maximise this modularity score to identify clusters where nodes are more densely connected to each other than to the rest of the network. This method allows for the identification of clusters of people in a social network who are connected through mutual relationships and by referring to the activities of the same users (e.g., by citing them or engaging in discussions about them).

We assumed that the minimum size of the group that allows for the analysis was 150 users. In terms of understanding the network graph, the results of this study were derived using the methodology developed in previous research [100]. We then constructed a sample of 200 posts, consisting of the 20 most re-posted posts from each of the 10 months of measurement (starting from March 2022), and used this sample for stance and frame detection.

Although the qualitative analysis focused on a selected sample of 200 posts, the analytical impact of this sample is significantly amplified by its reach within the full dataset. We initially tested different sampling thresholds – sets of 10, 20, and 30 posts per month – to assess the relationship between post popularity and representativeness. However, when the sample exceeded 20 posts per month, the additional items displayed a markedly lower number of reposts, which reduced the internal homogeneity of the dataset. Therefore, a threshold of 20 top-performing posts per month was adopted as optimal for maintaining both analytical consistency and representativeness. As shown in Table 3, these 200 posts generated a total of 22,772 reposts, accounting for approximately 41.4% of all collected items.

This clearly shows that the selected sample is not only analytically relevant but also representative of the discourse’s most widely circulated and influential content. In highly asymmetric communication ecosystems such as X/Twitter, a small number of posts often account for a disproportionate share of public attention and engagement. Consequently, focusing on the most popular posts enables the identification of dominant frames, emotionally resonant narratives, and politically salient attitudes shaping public discourse during the analysed period. This approach has been successfully applied in previous studies examining viral content and high-engagement posts as a proxy for public opinion trends [101,102].

Stance detection was used to help us understand how people expressed their opinions, attitudes, and emotions toward the topic of Ukrainian migrants in Poland. Due to the fact that none of the methods of automatic content analysis based on natural language processing produced satisfactory results, we decided to analyse the posts manually [103].

Guidelines provided to the annotators for our dataset are following the model described in Mohammad et al. [104]. The coding was confirmed by three annotators, all of whom were members of the author team. Five cases of disagreements were discussed and resolved by voting. The annotators were asked to answer two questions. The first question required selecting the stance category from one of four classes: in favour of migrants (F), against migrants (A), neutral (N), or none of the above (NA). In order to clarify the scope of each class, possible cases that apply to that particular class were provided within the instructions. In the second question, annotators were asked to determine whether the focus of the post was the stance target, an entity other than the target, or whether the post had any focus at all. At the end of the annotation procedure, the number of posts annotated with None of the Above stance was found to be less than 1%, and therefore, the third and the fourth classes were combined into one stance class as Neutral (N). Each stance was assigned a value (A = 0; N = 0,5, F = 1) to calculate the average stance for frames and groups. Once the stance was identified, the conversion rate for each stance was calculated as the number of reposts divided by the number of posts assigned to that stance. The number of reposts reflects the reach of a post’s dissemination [101], and dividing it by the number of posts within a given stance enables an analysis of the average reach of each stance. These analyses constituted the initial step towards a more comprehensive qualitative analysis conducted in accordance with the guidelines of frame analysis.

Previous research on framing has proposed numerous typologies of frames [11]. The typology employed in our analysis distinguishes between generic and issue-specific frames. Generic frames are applicable across various contexts, facilitating comparative studies. In this study, the selection of generic frames was based on the framework developed by Semetko and Valkenburg [68], who identified the following categories: (a) conflict, (b) human interest, (c) economic, (d) morality, and (e) responsibility (Table 1).

**Table 1:** Generic Frames.

Generic frame	Question used to identify the frame	Frame code
Conflict	Does the story reflect disagreement between parties, individuals, or groups?	CON
Human interest	Does the story emphasize how individuals and groups are affected by the issue or problem?	HI
Economic	Does the story mention financial losses or gains, either in the present or future?	ECON
Morality	Does the story contain any moral message?	MOR
Responsibility	Does the story suggest that a certain level of government is responsible for the issue or problem?	RES

Source: own elaboration based on Semetko and Vakenburg [68]

Issue-specific frames, in contrast, pertain to the specific context and issue being framed [50,61,105]. They are more detailed and authoritative in nature, rendering them unsuitable for comparative studies. In this study, we identified five issue-specific frames related to the representation of Ukrainians in Poland in the following contexts: (a) migration movements, (b) characteristics of immigrants, (c) assistance provided to Ukrainians and the Polish government's policies towards Ukrainian residents, (d) World War II, and (e) international relations (Table 2).

**Table 2:** Issue-specific frames.

Issue-specific frame	Question used to identify the frame	Frame Code
Migration flows	Does the story concern the influx or outflow of Ukrainians?	FLOW
Migrant features	Does the story highlight the characteristics of migrants?	FEAT
Helping Ukrainians, bottom up (society) and top down (internal policy)	Does the story discuss grassroots and governmental support?	HELP
Second World War	Does the story relate to the Second World War?	WWII
International relations	Does the story emphasise the impact of the issue/problem on international relations?	INT

Source: own elaboration.

To identify the frames, we employed qualitative content analysis. The data came from the assessments of three independent coders. Both generic and issue-specific frames were coded based on the questions outlined in Tables 1 and 2. When identifying generic frames, coders supplemented the questions in Table 1 with an extended set of questions developed by Semetko and Valkenburg for each frame [68]. Tables 1 and 2 present the complete coding scheme applied in this study. The criteria for identifying issue-specific frames were derived from a preliminary analysis (see S1 Table for the list of questions assigned to each frame). Furthermore, annotators had the option to assign a post to multiple frames if necessary.

In interpreting the assignment of texts to specific frames, we also incorporated insights from Hameleers [106] on the interplay between textual and visual elements in social media posts. This approach acknowledged that visual information, when paired with textual captions or comments, can be intentionally decontextualized or reinterpreted to construct and legitimize specific partisan narratives.

The coding of frames was conducted exclusively by members of the author team, which comprised a multidisciplinary group consisting of a political scientist, a political sociologist, and a computer scientist specializing in social informatics. Prior to the formal coding stage, the team undertook a series of preparatory procedures to ensure coding consistency. These included a joint review of a randomly selected subset of tweets, during which the coders collaboratively applied the preliminary coding scheme and discussed any divergent interpretations. This calibration phase was preceded by a training session aimed at aligning conceptual understandings of the frame categories and establishing clear operational definitions. A short pilot study involving 20 tweets was conducted to refine both the coding guidelines and the coders’ application of them. Following these preparatory steps, the coders proceeded to independently annotate the dataset.

To assess intercoder reliability before consensus-based reconciliation, we calculated Krippendorff’s alpha for the full set of initial codings across all three coders. The resulting coefficient was 0.92, indicating a high level of agreement. This metric reflects the level of consistency prior to any resolution of discrepancies. Only five instances required further discussion to reach consensus. No majority vote was necessary, as consensus was achieved in all cases through deliberation.

Following the identification of frames, the conversion rate for each frame was calculated, and a hierarchical cluster analysis was conducted using Ward’s method [107]. The frame conversion ratio was determined by dividing the number of reposts by the number of posts assigned to the frame. A hierarchical cluster analysis, using squared Euclidean distance as a measure of similarity, was performed to examine the co-occurrence of frames. The results were presented in a dendrogram.

X/Twitter tends to be more actively used by politically engaged individuals, journalists, and public figures, which may lead to the overrepresentation of elite or activist perspectives. Engagement metrics such as repost counts may therefore be influenced by platform-specific structural factors, including network effects, coordinated activity, and the increased visibility of accounts with large follower bases. Accordingly, the interpretation of engagement metrics was conducted with particular caution. Higher conversion rates of posts expressing a positive stance were not treated as indicators of universal public endorsement but were interpreted with reference to the political context of the analysed period and to platform-specific visibility structures. It is also important to note that X/Twitter users do not constitute a representative sample of the Polish population, and the opinions expressed on the platform cannot be directly generalised to overall public opinion. Social media discourse is shaped by platform-specific demographics, user behaviour norms, and the amplification of particular voices through algorithmic recommendation systems.

All data collection and analysis procedures were conducted in full compliance with X/Twitter’s Developer Policy and Terms of Service. Data collection (2022) complied with the Twitter Developer Agreement effective October 10, 2022 and the corresponding Twitter Developer Policy in force at that time. Subsequent data processing and analysis (2023–2024) were conducted on the previously collected dataset and therefore did not involve additional data acquisition following the Developer Agreement update effective January 19, 2023. The dataset does not include full tweet texts, user handles, avatar URLs, or direct links to individual tweets. To prevent unauthorized redistribution of content or exposure of personally identifiable information, we applied a strict data cleaning protocol: (1) all full tweet texts were removed from the dataset and from any in-text examples or tables; (2) user handles and profile image links were anonymized or excluded, with the exception of accounts belonging to institutions or public figures, as described later in the text; and (3) only Tweet IDs and, where analytically necessary, anonymised User IDs were retained. Instructions for data rehydration using the Twitter API are provided to ensure that any retrieval of content complies with the platform's current access policies.

## 5. Results

### 5.1. Overview of stance and frames in the sample based on Top-20 rankings

In the analysed sample of 200 posts, derived from the Top-20 monthly rankings over a 10-month period, a clear predominance of posts expressing a negative stance towards migrants (A) was observed, accounting for 60% of all posts. Posts with a neutral and positive stance account for 21.5% and 18.5% of the sample, respectively. This distribution results in an average stance score of 0.31 for the entire sample. On average, a single post generates 113.9 reposts. At the same time, we observe that posts with a negative stance—despite being the most numerous in the sample—have the lowest conversion rate. In contrast, posts with a positive stance, which are the least frequent, exhibit the highest conversion rate (Table 3). This finding aligns with previous studies on X/Twitter, which indicate that posts expressing positive emotions are more likely to be reposted [108,109]. Interestingly, as we will demonstrate later, the negative posts in our sample are primarily authored by a specific user group and generate the lowest conversion rates. Posts from this group consistently undermined expressions of solidarity and lowered the average stance score across both framing systems. This phenomenon will be explored in further detail in the discussion section.

**Table 3:** Stance and conversion levels across user groups, based on the sample of Top-20 most reposted posts for each month over a 10-month period (N = 200). Stance categories include: in favour of migrants (F), against migrants (A), and neutral (N).

	STANCE				Average Stance
	A	N	F	TOTAL	
Reposts	11762	4584	6426	22772	0,31
Posts	120	37	43	200	
Conversion	98	123,9	149,4	113,86	

As shown in Table 4, the sample is dominated by posts classified under the *conflict* (CON) and *helping Ukrainians* (HELP) frames. The highest conversion rates are observed for the *human interest* (HI), *morality* (MOR), and *international relations* (INT) frames, whereas the lowest conversion rates are found in the *Second World War* (WWII), *economy* (ECON), *migration flows* (FLOW), and *conflict* (CON) frames. Interestingly, the *human interest* (HI) frame generates the highest reach (i.e., conversion rate) despite comprising a relatively small number of posts, whereas the *conflict* (CON) frame, despite having the largest number of posts, exhibits a low reach. Stance detection within the frames reveals that negative posts dominate in the majority of categories. The only exceptions, where positive posts prevail, are the *human interest* (average stance = 0.85) and *international relations* (average stance = 0.57) frames.

**Table 4:** Conversion and stance for frames, based on the sample of Top-20 most reposted posts for each month over a 10-month period (N = 200). Generic frames: Conflict (CON), Human Interest (HI), Economic (ECON), Morality (MOR), Responsibility (RES). Issue-specific frames: Migration Flows (FLOW), Migrant Features (FEAT), Helping Ukrainians (HELP), Second World War (WWII), International Relations (INT).

	GENERIC FRAMES					ISSUE-SPECIFIC FRAMES				
CON	RES	HI	MOR	ECON	HELP	INT	FEAT	WWII	FLOW
Reposts	10055	5646	5189	4043	4038	9978	7596	8228	1934	1301
Posts	100	52	31	29	42	93	61	69	20	13
Conversion	100,6	108,6	167,4	139,4	96,1	107,3	124,5	119,3	96,7	100,1
Average stance	0,15	0,1	0,85	0,17	0,19	0,18	0,57	0,27	0	0,35

Notably, this finding aligns with previous research on generic frames in news coverage of the U.S. immigration debate during election years, which found that the *human interest* frame predominantly contained pro-immigrant statements. The same study also demonstrated that the *conflict* frame was the most frequently employed in discourse about migrants, a pattern that is also evident in our sample [63].

### 5.2. Groups and frames

**Fig 1:**
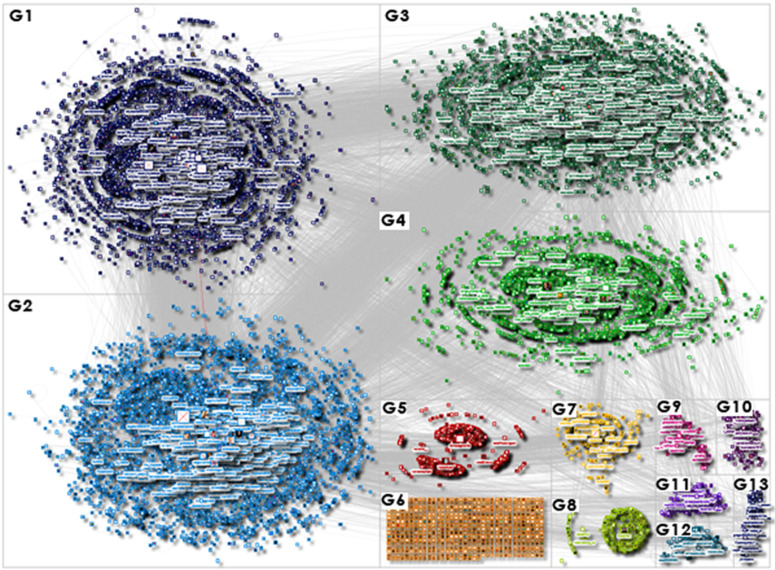
Social network graph of users grouped in the 13 largest groups (N = 19,462). The most significant groups include: G1 – Pro-Government Conservatives; G2 – Nationalist Opposition; G3 – Independent Commentators; G4 – Liberal Opposition and Media.

**Fig 2:**
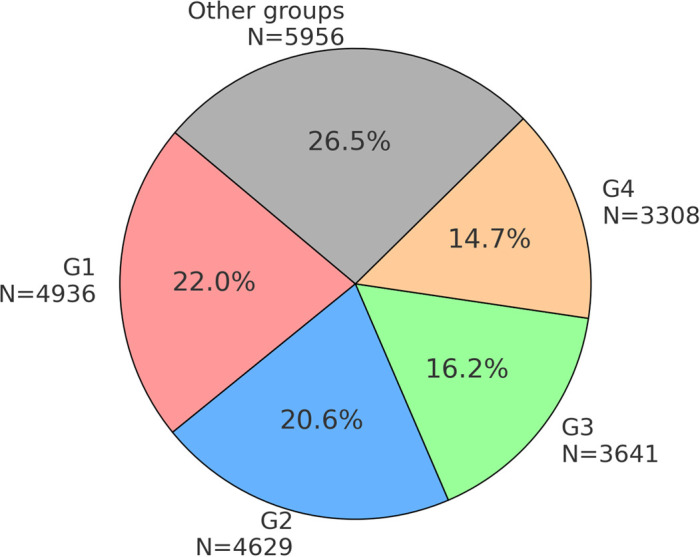
User distribution across identified groups: G1 (Pro-Government Conservatives); G2 (Nationalist Opposition); G3 (Independent Commentators); G4 (Liberal Opposition and Media).

For user clustering, we employed the Clauset–Newman–Moore algorithm, which identifies clusters or communities in large networks by focusing on hierarchical agglomeration based on the underlying network structure. The NodeXL software enabled us to cluster users into several groups based on their interactions, specific mentions, reposts, and replies. This analysis resulted in the identification of 13 user-groups that met the criterion of a minimum of 150 users, collectively representing approximately 86.62% users of the entire debate (N = 19,462). Fig 1 below illustrates the social network graph of users grouped into these 13 largest clusters. Among these, we identified the four most significant clusters, designated G1–G4, which accounted for 73.49% of the total users (N = 16,514). These four groups were subsequently subjected to further analysis. The distribution of users across specific groups is presented in Fig 2. The “Other groups” category includes the remaining 591 user groups, excluding G1–G4.

The differences between the identified groups become evident when analysing the users who are most frequently mentioned by other participants in the debate within each group. Mentioning is a distinctive feature of communication on the X/Twitter platform, characterised by the addition of a reference to another user within a post. Table 5 below presents the top-mentioned users for groups G1–G4.

**Table 5:** The ranking of the top-mentioned users in groups: G1 (Pro-Government Conservatives); G2 (Nationalist Opposition); G3 (Independent Commentators); G4 (Liberal Opposition and Media).

Top Mentioned in G1	Count	Top Mentioned in G2	Count
Cezarykrysztopa	719	[User T1]	2667
Dawidwildstein	595	[User 05]	755
Morawieckim	527	Andrzejduda	500
[User 01]	325	Jacekmiedlar	481
[User 02]	307	[User 06]	475
Jacekpiekara	269	[User 07]	452
bielsat_pl	263	marcinrola89	446
[User 03]	253	[User 08]	434
Melnykandrij	234	[User 09]	390
[User 04]	223	Pisorgpl	387
Top Mentioned in G3	Count	Top Mentioned in G4	Count
lkwarzecha	128	giertychroman	710
konfederacja_	114	[User 14]	297
jkmmikke	89	donaldtusk	291
grzegorzbraun_	83	[User 15]	289
[User 10]	71	[User 16]	266
[User 11]	66	jhaszczynski	257
[User 12]	64	[User 17]	253
[User 13]	63	lis_tomasz	247
warnewspl1	61	arkadiuszmyrcha	232
Wyrwal	61	pawelkowalpl	224

**Fig 3:**
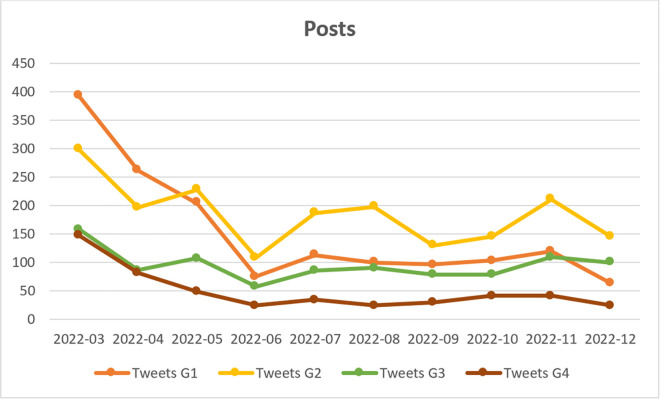
Posts published in groups: G1 (Pro-Government Conservatives); G2 (Nationalist Opposition); G3 (Independent Commentators); G4 (Liberal Opposition and Media) (N = 4813).

**Fig 4:**
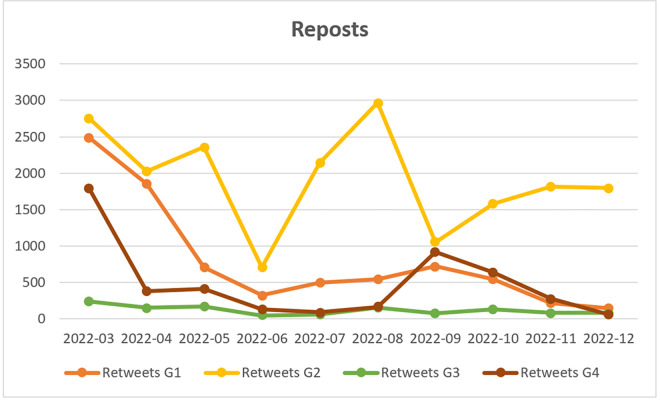
Reposts published in groups: G1 (Pro-Government Conservatives); G2 (Nationalist Opposition); G3 (Independent Commentators); G4 (Liberal Opposition and Media) (N = 33,311).

**Fig 5:**
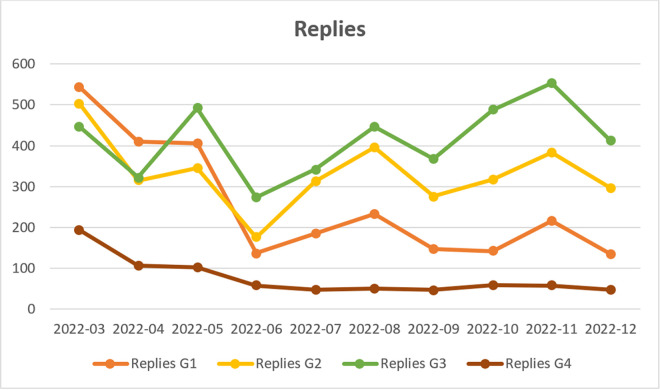
Replies published in groups: G1 (Pro-Government Conservatives); G2 (Nationalist Opposition); G3 (Independent Commentators); G4 (Liberal Opposition and Media) (N = 10,792).

In Group G1, the most frequently mentioned individuals include journalists, politicians, and influencers associated with the right-wing Law and Justice Party, which governed Poland from 2016 to 2023. Notable figures mentioned in this group include Dawid Wildstein (former deputy director of public television), Cezary Krysztopa (a satirical cartoonist), and Mateusz Morawiecki (then Prime Minister). Other notable users include Jacek Piekara, a prominent fantasy author and right-wing commentator; the official profile of the publicly funded Belsat television channel; and Andriy Melnyk, serving at the time as Ukraine’s Deputy Foreign Minister. The identities of all remaining users have been anonymized. The analysis of user activity in G1 (Figs 3–5) indicates that engagement was highest during the first three months of the study period, after which it declined significantly. As shown in Table 6, out of the 200 posts which were included in the monthly Top-20 rankings, 35 were created by G1 users. Posts expressing a positive stance towards Ukrainian migrants dominate within this group (average stance = 0.8). Despite their relatively small number, these posts generate high conversion rates. According to Park & Kaye [102], this pattern is characteristic of accounts managed by mass media, established journalists, and politicians associated with government circles, whose posts on the X/Twitter platform tend to reach a wide audience. Posts from this group primarily report on statements and actions taken within the political environment governing Poland at the time. As shown in Table 7, an analysis of generic frames in G1 reveals that the *responsibility* (RES) and *economic* (ECON) frames appear infrequently in posts from this group and exhibit low conversion rates. The *morality* (MOR) frame, although containing a small number of posts, generates the highest conversion rate among all frames in G1. Posts within this frame highlight positive moral evaluations of the actions of both Ukrainians and Poles. Among the analysed posts, the *human interest* (HI) and *conflict* (CON) frames are the most prevalent. Posts in these categories achieve moderately high conversion rates. The human interest frame includes posts that emphasise the suffering of Ukrainians and encourage sympathy, whereas the conflict frame focuses on tensions with opponents of Ukrainian presence in Poland and highlights the perceived positive outcomes of the migration crisis.

**Table 6:** Distribution of stance categories across user groups, based on the sample of Top-20 most reposted posts for each month over a 10-month period (N = 200). Stance categories include: in favour of migrants (F), against migrants (A), and neutral (N). The groups include: G1 – Pro-Government Conservatives; G2 – Nationalist Opposition; G3 – Independent Commentators; G4 – Liberal Opposition and Media.

		STANCE				Average Stance
		A	N	F	TOTAL	
G1	Reposts	414	974	3542	4930	0,8
	Posts	3	8	24	35	
	Conversion	138	121,6	147,6	140,9	
G2	Reposts	11188	1591	205	12984	0,1
	Posts	114	16	2	132	
	Conversion	98,1	99,4	102,5	98,4	
G3	Reposts	41	95	N/A	136	0,4
	Posts	1	3	N/A	4	
	Conversion	41	31,7	N/A	34	
G4	Reposts	28	1298	1972	3298	0,83
	Posts	1	5	14	20	
	Conversion	28	259,6	140,9	164,9	

**Table 7:** Frames in groups, based on a sample of the Top-20 most reposted posts for each month over a 10-month period (N = 200). Generic frames: Conflict (CON), Human Interest (HI), Economic (ECON), Morality (MOR), Responsibility (RES). Issue-specific frames: Migration Flows (FLOW), Migrant Features (FEAT), Helping Ukrainians (HELP), Second World War (WWII), International Relations (INT). The groups include: G1 – Pro-Government Conservatives; G2 – Nationalist Opposition; G3 – Independent Commentators; G4 – Liberal Opposition and Media.

		GENERIC FRAMES					ISSUE-SPECIFIC FRAMES				
		CON	RES	HI	MOR	ECON	HELP	INT	FEAT	WWII	FLOW
G1	Reposts	1451	131	2414	786	93	1070	3136	1401	401	234
	Posts	11	2	17	3	1	10	22	9	2	2
	Conv.	131,9	65,5	142	262	93	107	142,6	155,7	200,5	117
G2	Reposts	7160	5172	558	3078	3132	7684	1683	5556	1533	1000
	Posts	81	44	3	25	35	72	22	52	18	9
	Conv.	88,4	117,6	186	123,1	89.5	106,7	85,2	106,9	85,2	111,1
G3	Reposts	41	77	N/A	N/A	24	94	24	18	N/A	53
	Posts	1	2	N/A	N/A	1	2	1	1	N/A	1
	Conv	41	38,5	N/A	N/A	24	47	24	18	N/A	53
G4	Reposts	1403	161	954	179	775	949	1849	747	N/A	N/A
	Posts	7	2	6	1	4	6	10	5	N/A	N/A
	Conv.	200,4	80,5	159	179	193,8	158,2	184,9	149,4	N/A	N/A

G1 posts were also analysed in terms of issue-specific frames. The largest number of posts fall under the *international relations* (INT) and *helping Ukrainians* (HELP) frames, with the former exhibiting a high conversion rate and the latter a moderate one. The high conversion rate for the international relations frame is likely due to the rapid pace of international political events, which encouraged the migration of political discourse to the X/Twitter platform. Surprisingly, the *helping Ukrainians* frame, despite containing a relatively large number of posts, has the lowest conversion rate. This is likely because both government and non-government actions related to refugee assistance were frequently reported across various media channels, leading to an oversaturation of such information. The least frequently used frames are *migration flows* (FLOW) and *Second World War* (WWII), each represented by only two posts. While the *migration flows* frame has an average conversion rate, the *Second World War* frame records the highest conversion rate among all issue-specific frames. Posts in this frame typically discuss anniversaries of historical events, focusing on Ukrainian culpability and historical policy mistakes. The high conversion rate of this frame is likely due to its appeal among nationalist-leaning users, as reflected in the activity of the G2 group.

In contrast to Group G1, Group G2 predominantly mentions individuals associated with nationalist political circles, including the controversial former priest and internet figure Jacek Międlar and the radical journalist Marcin Rola. Government-affiliated accounts, such as that of Polish President Andrzej Duda and the official profile of the Law and Justice Party (“pisorgpl”), are primarily mentioned as targets of criticism. As shown in Fig 4, user activity in Group G2 is particularly evident in content reposting, peaking in July and August 2022, which is related to the day of remembrance of the Slaughter of Volhynia, which falls on 11 July. Similarly, in terms of original posts (Fig 3), these users begin to dominate in mid-2022. However, they are significantly less active in engaging in polemical discussions (Fig 5). G2 users are not part of the mainstream political and opinion-forming landscape, which likely explains why their posts achieve lower reach compared to those of the G1 and G4 groups. However, it is important to highlight that G2 users display a strong focus on the influx and presence of Ukrainians in Poland, producing 132 of the 200 posts in the Top-20 monthly rankings sample. While the conversion rate of these posts is lower than that of G1 and G4 users, it remains higher than that of G3. A key characteristic of G2 discourse is its overwhelmingly negative stance towards Ukrainian migrants (average stance = 0.1). Generic frame analysis reveals that G2 posts predominantly employ the *conflict* frame (N = 81), portraying conflicts related to the presence of Ukrainian migrants in Poland in a negative light and focusing on controversial situations. However, the high volume of posts using this frame does not translate into high conversion rates. This type of content was reluctantly reposted, possibly due to prevailing political correctness in the early months of the war, which G2 users attempted—unsuccessfully—to challenge. The second and third most frequently used frames in G2 posts are the *responsibility* and *economic* frames, both of which exhibit slightly higher conversion rates. Posts using the *responsibility* frame assign blame to political leaders for the negative consequences of accepting Ukrainian migrants, while the *economic* frame posts highlight the adverse effects of the refugee crisis on the economy and market relations. G2 users employ the *morality* frame less frequently, but when they do, it is used to highlight perceived unethical behaviour by both Ukrainians and Poles. This frame also generates a higher conversion rate than the *conflict* frame. Notably, the *human interest* frame is the least frequently used in G2 posts, despite having the highest conversion rate. This suggests that posts using the human interest frame tend to be more widely shared. A likely explanation for this trend is the specific rhetorical construction of these posts, which follow a “sandwich” format—combining expressions of dislike and aggressive commentary about Ukrainians with an empathetic description of the challenges faced by both Poles and Ukrainians. This structure may contribute to their higher shareability, as it enables subtle expressions of discontent while still appealing to wider audiences.

An interesting observation regarding the activity of the G2 group is the behaviour of the user labelled as “User T1”, who appears in Table 5 as the most frequently mentioned debate participant. Our analysis showed that from the creation of the account on 6th June 2021 to the end of the analysed period on 23rd May 2023, the user generated a total of 13,581 posts over 716 days, averaging 18.97 posts per day. Such an exceptionally high posting frequency, combined with the highly emotional and polarising content of the posts, aligns with characteristics commonly associated with internet trolling. Similar high-frequency posting patterns are often linked to disinformation campaigns, automated bot activity, or co-ordinated influence operations, rather than genuine individual engagement [110]. A review of the content posted by this user indicates that it is almost entirely repetitive, emotional, and highly polarised, seemingly designed to dominate discourse and hinder meaningful engagement from other users. These characteristics align with the hallmarks of internet trolling, as identified by Scriven [111].

While we recognise that the intent behind such accounts may not reflect genuine individual engagement, their content nonetheless generated measurable reactions among other X/Twitter users, including reposts and replies. For this reason, and in line with our aim of capturing the full spectrum of discourse visible to platform users at the time, we treated posts from such accounts in the same analytical manner as other contributions. This decision was based on the premise that highly polarised or manipulative content can influence the tone and direction of online debates, regardless of its origin or authenticity. By including these posts, we sought to document and quantify their potential impact, thereby contributing to the broader understanding of how such discursive interventions operate and how they might be more effectively identified and addressed in future research on public opinion in digital environments.

An analysis of issue-specific frames revealed that G2 users frequently employed frames relating to *helping Ukrainians* (HELP) and migrant features (FEAT) to criticise the provision of aid to Ukrainians, as well as to highlight negative attributes of migrants. These frames achieved relatively high levels of engagement. The second most commonly used frame was that of *international relations* (INT), which was deployed to underscore the negative aspects and contradictions of Poland’s support for the Ukrainian state and its citizens. However, this frame was characterised by low engagement. A similar pattern emerged in relation to posts employing the *Second World War* (WWII) frame. The G2 group was responsible for the majority of posts within this category, focusing on the resentment expressed by a segment of the Polish population towards crimes committed by Ukrainian militias during the war – crimes that, according to this group, have yet to be fully investigated or acknowledged. However, these posts did not attract significant engagement in the form of reposts. By contrast, the highest level of engagement was recorded in the *migration flows* (FLOW) frame. This category contained relatively few posts in which G2 members combined descriptions of population movement with narratives portraying Ukrainians in a negative light – specifically, framing migration as either an attempt to evade conscription or a deliberate choice to seek a more comfortable life in exile, despite large parts of Ukraine remaining unaffected by war.

Group G3 exhibits distinct characteristics compared to the other groups, primarily due to the significantly higher number of replies and the lower number of reposts (Fig 4,5). Members of this group emerge as discussants who are less inclined to amplify others’ posts and are instead more focused on expressing their own views through replies. For this reason they were designated as “Independent Commentators.” The data presented in Table 6 indicates that the number of posts and reposts in G3 within the monthly Top-20 rankings was considerably lower than in other groups, suggesting that no single authority figure dominated the discourse. Notably, the right-wing journalist Łukasz Warzecha (“lkwarzecha”), controversial right-libertarian politicians, such as Janusz Korwin-Mikke (“jkmmikke”) and Grzegorz Braun (“grzegorzbraun_”), as well as the official profile of the far-right political party Konfederacja are often mentioned in the group, yet they frequently become subjects of criticism rather than authoritative voices. Other accounts belonging to public figures included that of M. Wyrwał, an investigative journalist for the news portal Onet in Ukraine, as well as the WarNewsPL service, which specializes in reporting on armed conflicts. However, as we observed, their popularity was significantly lower compared to the other identified groups. The sample of monthly top-20 posts contains only four posts from G3 users, which precludes a more detailed analysis of their opinions. However, these posts received a sufficient number of reposts to be included in the dataset. Similar to the G2 group, G3 does not include politicians from the most influential political parties or journalists affiliated with mainstream media, which explains the limited engagement (conversion) of its posts. The analysed dataset includes three neutral posts and one negative post. The negative post employs the *conflict* and *migrant features* frames, warning against the so-called “Ukrainianisation of Poland” (slogan used by far-right representatives), and the perceived hasty integration of Ukrainians into Polish political life. An analysis of generic frames reveals the absence of posts using *human interest* and *morality* frames. There is a single instance of the *conflict* and *economy* frames, alongside two occurrences of the *responsibility* frame. An examination of issue-specific frames indicates the absence of posts employing the *Second World War* frame, while other frames were used sporadically and reflected a neutral stance towards Ukrainians.

In group G4, the most frequently mentioned individuals were affiliated with the liberal political opposition at the time, including former (and, as of 2025, current) Prime Minister Donald Tusk, politician Roman Giertych, members of parliament Arkadiusz Myrcha and Paweł Kowal, as well as journalists Tomasz Lis (former editor-in-chief of *Newsweek Polska*) and Jerzy Haszczyński (*Rzeczpospolita*). The G4 group comprises prominent journalists and well-known politicians affiliated with the main parliamentary opposition party; accordingly, it has been labeled as “Liberal Opposition and Media.” Interestingly, although this group had only slightly fewer users than G1–G3 groups (see Fig 2), they were significantly less active in terms of posting and replying (Figs 3 and 5). They show increased activity in reposting in September, when the anniversary of Germany and Russia's invasion of Poland from September 1939 takes place, prompting support for Ukraine and Ukrainians, and reflections on the international situation. As in the case of group G1, the G4 posts in Top-20 monthly rankings overwhelmingly expressed a positive stance towards Ukrainians in Poland (average stance = 0.83). Although relatively few in number (N = 20), these posts achieved significant reach, as measured by the number of reposts. An analysis of generic frames indicates that G4 users most frequently employed the *conflict* and *human interest* frames, a pattern similar to that observed in G1. The *conflict* frame exhibited the highest conversion rate, while the *human interest* frame also achieved relatively high engagement. The *conflict* frame primarily referenced internal Polish political disputes, particularly those involving opposition to refugee acceptance. Meanwhile, the *human interest* frame contained posts aimed at eliciting empathy for both Ukrainians and Poles. The *economic* frame displayed a comparable conversion rate but was used in fewer posts. The *moral* frame was the least represented, with only one post in the Top-20 rankings, highlighting the positive behaviour of Ukrainians living in Poland. Similarly, the *responsibility* frame contained very few posts. Notably, one of the two posts within this category was ironic, not criticising the government directly but rather mocking far-right and nationalist critiques of governmental actions. This suggests a temporary alignment between the liberal political opposition and Poland’s ruling circles in their response to the refugee crisis.

An issue-specific analysis revealed an absence of the *migration flows* and *Second World War* frames. The *international relations* frame, which achieved the highest conversion rate, was the most frequently employed, appearing in 10 out of 20 posts by G4 users. This reflects their strong interest in the topic. In addition to addressing informational aspects, these posts highlight Poland’s actions on the international stage and critique certain aspects of the ruling party’s foreign policy. This focus is closely linked to the dynamic geopolitical shifts occurring during the early phase of the war.

### 5.3 Cluster analysis

**Fig 6:**
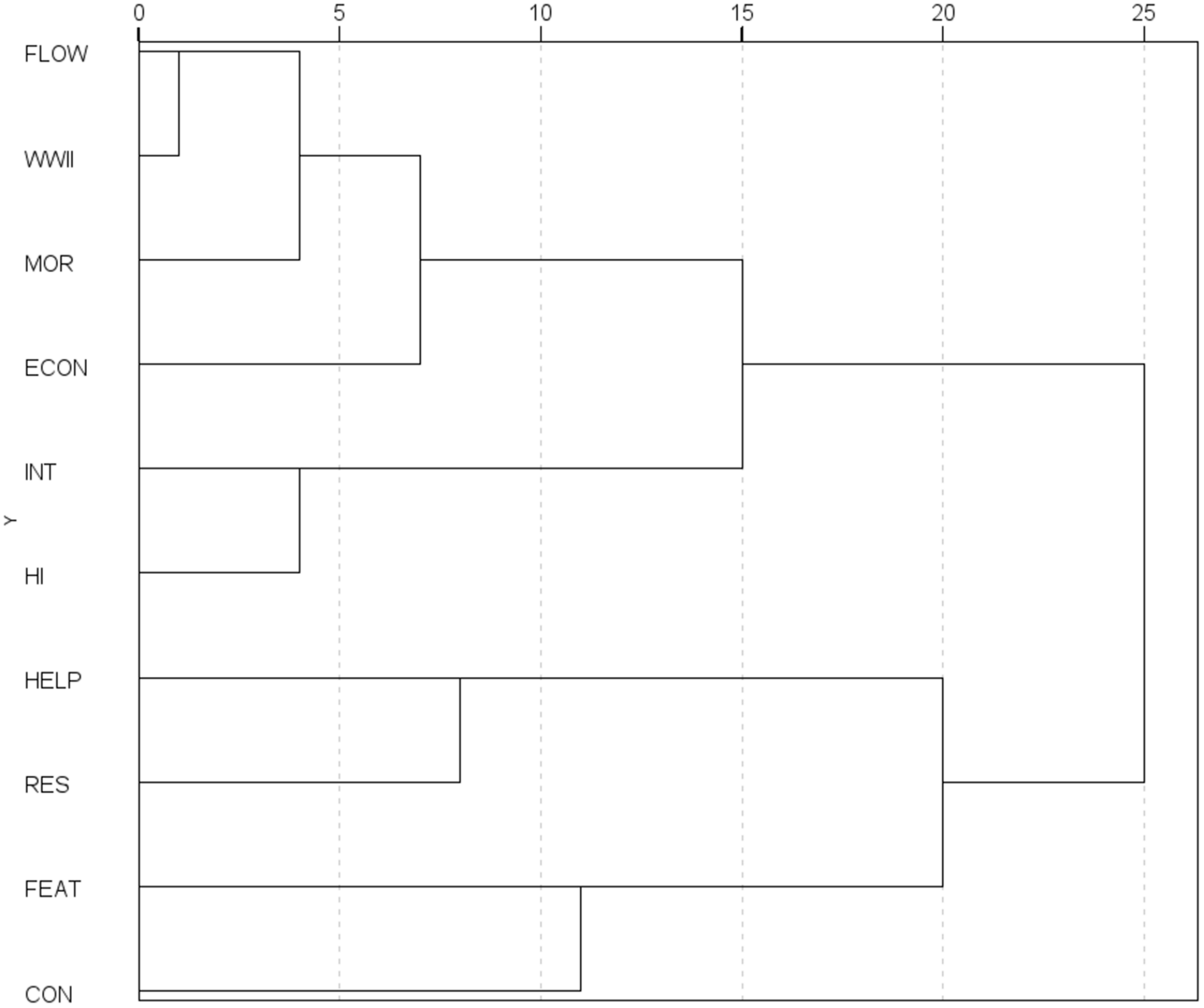
Dendrogram illustrating the results of a cluster analysis on frame co-occurrence. Generic frames: Conflict (CON), Human Interest (HI), Economic (ECON), Morality (MOR), Responsibility (RES). Issue-specific frames: Migration Flows (FLOW), Migrant Features (FEAT), Helping Ukrainians (HELP), Second World War (WWII), International Relations (INT).

The results of the hierarchical cluster analysis, conducted using Ward’s method to examine the co-occurrence of frames, are presented as a dendrogram (Fig 6). Four clusters of frames were identified in the sample. The first cluster comprises the *migrant features* (FEAT) and *conflict* (CON) frames, combining descriptions of social conflicts with portrayals of Ukrainian migrants. The *conflict* frame was widely employed across all groups, albeit in different contexts and with varying stances, encompassing both domestic conflicts within Poland and Polish-Ukrainian tensions.

The second cluster comprises a combination of the *human interest* (HI) and *international relations* (INT) frames, presenting an empathetic account of the impact of international events on the situation of both Ukrainians and Poles. This combination was characteristic of the G1 and G4 groups, which expressed positive attitudes towards refugees from Ukraine.

The third cluster consists of the *responsibility* (RES) and *helping Ukrainians* (HELP) frames, a combination predominantly found in the G2 group. This cluster framed assistance to Ukrainians through the lens of its negative consequences, attributing responsibility for them to those in power.

The fourth cluster encompasses posts that employ the *migrant flows* (FLOW) or *Second World War* (WWII) frames while simultaneously highlighting the negative economic or moral effects attributed to the presence of Ukrainians in Poland. This cluster reflects the publishing strategy of G2 users, who frequently voiced concerns about the influx of Ukrainians and invoked historical moments of tension between Poland and Ukraine.

## 6. Discussion

The results largely correspond to the assumptions derived from the literature review. The low number of posts employing the *human interest* frame is characteristic of social media, where the role of traditional newsrooms has diminished. In the analysed sample, the *conflict, responsibility, migrant features*, and *helping Ukrainians* frames were dominant. Posts employing the *economic* frame appeared slightly less frequently, which is also consistent with previous studies [74], as did those using the *international relations* frame, which was associated with commentary on the changing geopolitical situation accompanying the influx of Ukrainian war refugees into Poland.

Our findings support the crisis informatics thesis that, during crises, social media users tend to behave in a rational and information-seeking manner [31,32,39,40]. This is evidenced by the fact that posts authored by nationalist users (G2), which promoted negative attitudes toward refugees, achieved a lower conversion rate than those published by authors representing mainstream political groups (G1 and G4), despite their numerical predominance.

Posts expressing a positive stance achieved the highest average conversion rate; however, stance–group analysis shows that a substantial share of these high-performing posts originated from official accounts of politicians affiliated with the then-governing Law and Justice (PiS) party, as well as from state-aligned media actors, primarily clustered in group G1. These accounts benefit from elevated visibility and amplification across multiple media channels, which increases repost potential independently of message content. The positive stance observed in these posts largely mirrored the official political narrative of solidarity with Ukrainian refugees during the first year of the war, shaped by Poland’s geopolitical positioning and the prevailing public climate at the time. Consequently, higher conversion rates primarily reflect the interaction between political context and platform-specific visibility structures rather than the spontaneous distribution of attitudes across the broader population of X/Twitter users.

Our findings are consistent with recent British research, which indicates that on X/Twitter, the anti-immigrant community is more active in posting than the pro-immigration community [112]. They also align with other studies examining attitudes towards Ukrainian migrants expressed on the X/Twitter platform following 24 February 2022. Research on Spanish-language X/Twitter conducted in 2022 found that while posts about refugees generally conveyed a positive attitude toward Ukrainians, they were also characterized by hostility toward refugees from the Global South [12]. Additionally, user reactions to news articles about Ukrainian refugees were predominantly positive [113]. This pattern reflects a broader tendency toward greater acceptance of migrants from culturally similar backgrounds, a phenomenon observed beyond social media platforms as well [113–115]. Conversely, a study on German-language X/Twitter reported lower levels of solidarity toward refugees in 2022 compared to 2015, suggesting that experiencing a second large wave of refugees within a short period may diminish public solidarity [116]. However, this situation does not apply to Poland, which in 2022 experienced its first major wave of refugees since the end of World War II.

The proposed method demonstrates how methodological triangulation can enhance frame analysis in the study of crisis-related communication on social media. While triangulation itself is not a new approach, its adaptation to the X/Twitter environment during war and social crisis provides a novel analytical perspective: one that captures how ruling elites and their challengers seek to strengthen or weaken various patterns of social solidarity. The method enables researchers to efficiently reduce the vast universe of posts to a manageable corpus using a transparent quantitative criterion (number of reposts). This makes it possible to conduct systematic analyses even under resource constraints, while minimizing the problem of uncontrolled skew that often affects small qualitative samples. The combination of stance detection, generic and issue-specific frames offers a rapid yet accurate insight into how interpretative frames are used in highly visible posts that shape public opinion and are subsequently amplified by traditional media, which increasingly adopt their agenda-setting functions. However, the method may be less effective when applied to social media platforms with different user profiles and interaction dynamics. Future advances in automated qualitative analysis may enable the use of this approach on larger datasets, though this will not always be advisable: as the size of the corpus increases, so does its heterogeneity, which can only be mitigated by dividing the material into smaller, internally coherent subsets. This study does not fully encompass the entirety of the topic. In the next stages of our research, we plan to complement the sample analysis with a more in-depth examination of the chronology of frame and stance popularity and conversion by month, as well as the detection of echo chambers to determine how frequently posts are reposted outside their original groups. This is particularly significant, as several social media studies suggest that the popularity of far-right posts is largely confined to their own readership and does not extend beyond these circles [117], and that monitoring highly active online users may be a potential way to mitigate anti-immigration sentiments [112].

To conclude, our findings relate to a specific period of the war and the social processes associated with it. Poland and Ukraine share a common enemy, Russia, which has influenced mutual perceptions between Poles and Ukrainians, including war refugees. This may explain the lower conversion rates of posts expressing negative attitudes towards Ukrainians in our study. In the first year of the war, replying to or reposting negative content could have led to ostracism due to a prevailing sense of political unity. As of 2022, the majority of Ukrainian war refugees in Poland were women and children [118], as men were not officially permitted to cross the border. According to the stereotype content model, such groups are often perceived as warm and well-intentioned but incompetent—that is, unable to effectively translate their intentions into action [119,120]. This premise, along with cultural proximity, helps explain the high engagement with positive posts. As another study on social media has demonstrated, at the onset of the war, even those opposed to accepting refugees tended to avoid overt aggression and discrimination, instead resorting to more subtle expressions of discontent—an approach that evolved over time [47]. In the long term, institutional and social support for refugees may lead to their increasing reliance on the welfare system, potentially reshaping initial social attitudes and altering existing stereotypes. This shift could weaken positive perceptions of Ukrainians in Poland, highlighting the need for periodic follow-up studies.

## Supporting information

S1 Text Brief background on Polish-Ukrainian relations [8,121–129]. (DOCX)

S1 Table Criteria for identifying issue-specific frames. (DOCX)

## References

[1] Tschirky M, Makhortykh M. #Azovsteel: Comparing qualitative and quantitative approaches for studying framing of the siege of Mariupol on Twitter. Media, War & Conflict. 2023;17(2):163–78. doi: 10.1177/17506352231184163

[2] Dunn EC, Kaliszewska I. Distributed humanitarianism: Volunteerism and aid to refugees during the Russian invasion of Ukraine. American Ethnologist. 2023;50(1):19–29. doi: 10.1111/amet.13138

[3] Klaus W, Szulecka M. Departing or Being Deported? Poland’s Approach towards Humanitarian Migrants. Journal of Refugee Studies. 2022;36(3):467–88. doi: 10.1093/jrs/feac063

[4] Thérová L. Anti-immigration attitudes in contemporary Polish society: A story of double standards? Nationalities Papers. 2023;51(2):387–402. doi: 10.1017/nps.2022.71

[5] Office for Foreigners. Raport na temat obywateli Ukrainy (według stanu na dzień 31 stycznia 2024 r). Warszawa: Office for Foreigners. 2024. https://www.gov.pl/attachment/831fe2c9-ecbc-4c06-a9d5-7380e82457ea

[6] Duszczyk M, Górny A, Kaczmarczyk P, Kubisiak A. War refugees from Ukraine in Poland – one year after the Russian aggression. Socioeconomic consequences and challenges. Regional Science Policy & Practice. 2023;15(1):181–200. doi: 10.1111/rsp3.12642

[7] UNHCR. Operational Data Portal: Ukrainian Refugee Situation. https://data.unhcr.org/en/situations/ukraine. 2022. Accessed 2024 July 30.

[8] Statistics Poland. Bank Danych Lokalnych. Warszawa: Statistics Poland. 2024. https://bdl.stat.gov.pl

[9] Calderón CA, Blanco-Herrero D, Sánchez-Holgado P, Barradas-Gurruchaga A. Migrants and refugees on Twitter in Spain: A study employing automated analysis into the presence of hate and sentiment. Doxa Comunicación. 2023;38:369–89. doi: 10.31921/doxacom.n38a1734

[10] Arcila-Calderón C, Sánchez-Holgado P, Quintana-Moreno C, Amores J-J, Blanco-Herrero D. Hate speech and social acceptance of migrants in Europe: Analysis of tweets with geolocation. Comunicar: Revista Científica de Comunicación y Educación. 2022;30(71):21–35. doi: 10.3916/c71-2022-02

[11] de Saint Laurent C, Glaveanu V, Chaudet C. Malevolent Creativity and Social Media: Creating Anti-immigration Communities on Twitter. Creativity Research Journal. 2020;32(1):66–80. doi: 10.1080/10400419.2020.1712164

[12] Essalhi-Rakrak A, Pinedo-González R. #EspañaInvadida. Disinformation and hate speech towards refugees on Twitter: A challenge for critical thinking. EPI. 2023. doi: 10.3145/epi.2023.may.10

[13] Fuentes-Lara C, Calderón CA. Islamophobic hate speech on social networks: An analysis of attitudes to Islamophobia on Twitter. Revista Mediterránea de Comunicación. 2023;14(1):225–40. doi: 10.14198/MEDCOM.23044

[14] Kapidzic S, Frey F, Neuberger C, Stieglitz S, Mirbabaie M. Crisis communication on Twitter: Differences between user types in top tweets about the 2015 “Refugee Crisis” in Germany. International Journal of Communication. 2023;17:735–54.

[15] Koytak HZ, Celik MH. A Text Mining Approach to Determinants of Attitude Towards Syrian Immigration in the Turkish Twittersphere. Social Science Computer Review. 2022;41(2):608–25. doi: 10.1177/08944393221117460

[16] Martinez de Bartolome I, Rivera Martín B. Analysis of media discourse and social audiences on refugees in the conflict in Ukraine. Tripodos. 2023;53:03. doi: 10.51698/tripodos.2022.53.03

[17] Menshikova A, van Tubergen F. What Drives Anti-Immigrant Sentiments Online? A Novel Approach Using Twitter. European Sociological Review. 2022;38(5):694–706. doi: 10.1093/esr/jcac006

[18] Mulki H, Alabdullah S, Halil A, Al-Ali N, Kyriakidou M, Stavinoha L. Online toxicity against Syrians in Turkish Twitter: Analysis and implications. International Journal of Communication. 2023;18:28.

[19] Pérez-Curiel C, Garrote-Fuentes Á, Rivas-de-Roca R. EU and crisis management: Afghanistan and Ukraine on social media. Front Polit Sci. 2023;5. doi: 10.3389/fpos.2023.1138445

[20] Sánchez-Holgado P, Amores JJ, Blanco-Herrero D. Online Hate Speech and Immigration Acceptance: A Study of Spanish Provinces. Social Sciences. 2022;11(11):515. doi: 10.3390/socsci11110515

[21] Avraamidou M, Ioannou M. Migrants as ‘pawns’: Antimigrant debates on Twitter and their affinity to European border politics and discourses. European Journal of Cultural Studies. 2022;26(5):722–43. doi: 10.1177/13675494221120838

[22] Calderón CA, de la Vega G, Herrero DB. Topic Modeling and Characterization of Hate Speech against Immigrants on Twitter around the Emergence of a Far-Right Party in Spain. Social Sciences. 2020;9(11):188. doi: 10.3390/socsci9110188

[23] Froio C, Ganesh B. The transnationalisation of far right discourse on Twitter: Issues and actors that cross borders in Western European democracies. European Societies. 2018;21(4):513–39. doi: 10.1080/14616696.2018.1494295

[24] González-Baquero W, Amores JJ, Arcila-Calderón C. The Conversation around Islam on Twitter: Topic Modeling and Sentiment Analysis of Tweets about the Muslim Community in Spain since 2015. Religions. 2023;14(6):724. doi: 10.3390/rel14060724

[25] Tuñón-Navarro J, Bouzas-Blanco A. European far right on Twitter: Analysis of the digital communicative strategy of Vox and Lega during the 2014 and 2019 European elections. Revista Mediterránea de Comunicación/Mediterranean Journal of Communication. 2023;14(1):241–62. doi: 10.14198/MEDCOM.23334

[26] Walsh JP. Social media and border security: Twitter use by migration policing agencies. Policing and Society. 2019;30(10):1138–56. doi: 10.1080/10439463.2019.1666846

[27] Dimitrova D, Heidenreich T, Georgiev TA. The relationship between humanitarian NGO communication and user engagement on Twitter. New Media & Society. 2022;26(5):2514–34. doi: 10.1177/14614448221088970

[28] Henriksen FM, Kristensen JB, Mayerhöffer E. Dissemination of RT and Sputnik Content in European Digital Alternative News Environments: Mapping the Influence of Russian State-Backed Media Across Platforms, Topics, and Ideology. The International Journal of Press/Politics. 2024;29(3):795–818. doi: 10.1177/19401612241230281

[29] Morejón-Llamas N, Martín-Ramallal P, Micaletto-Belda JP. Twitter content curation as an antidote to hybrid warfare during Russia’s invasion of Ukraine. Profesional de la Información. 2022;31(3). doi: 10.3145/epi.2022.may.08

[30] Zhao B, Ren W, Zhu Y, Zhang H. Manufacturing conflict or advocating peace? A study of social bots agenda building in the Twitter discussion of the Russia-Ukraine war. Journal of Information Technology & Politics. 2023;21(2):176–94. doi: 10.1080/19331681.2023.2189201

[31] Reuter C, Kaufhold M. Fifteen years of social media in emergencies: A retrospective review and future directions for crisis Informatics. Contingencies & Crisis Mgmt. 2017;26(1):41–57. doi: 10.1111/1468-5973.12196

[32] Reuter C, Hughes AL, Kaufhold M-A. Social Media in Crisis Management: An Evaluation and Analysis of Crisis Informatics Research. International Journal of Human–Computer Interaction. 2018;34(4):280–94. doi: 10.1080/10447318.2018.1427832

[33] Emmer M. Soziale Medien in der politischen Kommunikation. Handbuch Soziale Medien. Springer Fachmedien Wiesbaden. 2016:81–99. doi: 10.1007/978-3-658-03765-9_5

[34] Gabel S, Reichert L, Reuter C. Discussing conflict in social media: The use of Twitter in the Jammu and Kashmir conflict. Media, War & Conflict. 2020;15(4):504–29. doi: 10.1177/1750635220970997

[35] El-Nawawy M, Khamis S. Egyptian Revolution 2.0: Political Blogging, Civic Engagement, and Citizen Journalism. Basingstoke: Palgrave Macmillan. 2013.

[36] Hafez K. Macht und Ohnmacht der Medien in Demokratisierungsprozessen: Lehren aus dem „Arabischen Frühling“. ZPol. 2014;24(3):341–51. doi: 10.5771/1430-6387-2014-3-341

[37] Kayyali D. Digital memory, evidence, and social media: lessons learned from Syria. Sociologica. 2022;16(2):253–9. doi: 10.6092/issn.1971-8853/15383

[38] Wolfsfeld G, Segev E, Sheafer T. Social Media and the Arab Spring: Politics Comes First. The International Journal of Press/Politics. 2013;18(2):115–37. doi: 10.1177/1940161212471716

[39] Reuter C, Heger O, Pipek V. Combining real and virtual volunteers through social media. In: Proceedings of the 10th International Conference on Information Systems for Crisis Response and Management (ISCRAM 2013), Baden-Baden, Germany, 2013:780–90.

[40] Vieweg S, Hughes AL, Starbird K, Palen L. Microblogging during two natural hazards events: What Twitter may contribute to situational awareness. In: Proceedings of the SIGCHI Conference on Human Factors in Computing Systems, 2010. 1079–88. doi: 10.1145/1753326.1753486

[41] Castles S, de Haas H, Miller MJ. The age of migration: International population movements in the modern world. 5th ed. Palgrave Macmillan. 2014.

[42] Dustmann C, Vasiljeva K, Piil Damm A. Refugee Migration and Electoral Outcomes. The Review of Economic Studies. 2018;86(5):2035–91. doi: 10.1093/restud/rdy047

[43] Esses VM, Hamilton LK, Gaucher D. The Global Refugee Crisis: Empirical Evidence and Policy Implications for Improving Public Attitudes and Facilitating Refugee Resettlement. Social Issues Policy Review. 2017;11(1):78–123. doi: 10.1111/sipr.12028

[44] Mendola D, Pera A. Vulnerability of refugees: Some reflections on definitions and measurement practices. International Migration. 2021;60(5):108–21. doi: 10.1111/imig.12942

[45] Pitropakis N, Kokot K, Gkatzia D, Ludwiniak R, Mylonas A, Kandias M. Monitoring Users’ Behavior: Anti-Immigration Speech Detection on Twitter. MAKE. 2020;2(3):192–215. doi: 10.3390/make2030011

[46] Calderón CA, Blanco-Herrero D, Valdez Apolo MB. Rejection and hate speech in Twitter: Content analysis of tweets about migrants and refugees in Spanish. Revista Española de Investigaciones Sociológicas. 2020;172:21–40. doi: 10.5477/cis/reis.172.21

[47] Yılmaz F, Elmas T, Eröz B. Twitter-based analysis of anti-refugee discourses in Türkiye. Discourse & Communication. 2023;17(3):298–318. doi: 10.1177/17504813231169135

[48] Blalock HA. Toward a theory of minority-group relations. New York: Wiley. 1967.

[49] The nature of prejudice. Cambridge: Perseus Books. 1954.

[50] De Vreese CH. News framing: Theory and typology. Information Design Journal Document Design. 2005;13(1):51–62. doi: 10.1075/idjdd.13.1.06vre

[51] Tuchman G. Making news: A study in the construction of reality. New York: Free Press. 1978.

[52] Cho SH, Gower KK. Framing effect on the public’s response to crisis: Human interest frame and crisis type influencing responsibility and blame. Public Relations Review. 2006;32(4):420–2. doi: 10.1016/j.pubrev.2006.09.011

[53] Entman RM. Framing: Toward Clarification of a Fractured Paradigm. Journal of Communication. 1993;43(4):51–8. doi: 10.1111/j.1460-2466.1993.tb01304.x

[54] Bryant J, Miron D. Theory and Research in Mass Communication. Journal of Communication. 2004;54(4):662–704. doi: 10.1111/j.1460-2466.2004.tb02650.x

[55] Cacciatore MA, Scheufele DA, Iyengar S. The end of framing as we know it … and the future of media effects. Mass Communication and Society. 2016;19(1):7–23. doi: 10.1080/15205436.2015.1068811

[56] Krippendorff K. Three concepts to retire. Annals of the International Communication Association. 2017;41(1):92–9. doi: 10.1080/23808985.2017.1291281

[57] Guenther L, Jörges S, Mahl D, Brüggemann M. Framing as a Bridging Concept for Climate Change Communication: A Systematic Review Based on 25 Years of Literature. Communication Research. 2023;51(4):367–91. doi: 10.1177/00936502221137165

[58] Musi E. Framing to Make an Argument: The Case of the Genocide Hashtag in the Russia-Ukraine war. Argumentation. 2024;38(3):269–88. doi: 10.1007/s10503-024-09632-1

[59] Nisch S. Invasion of Ukraine: Frames and sentiments in Zelensky’s Twitter communication. Journal of Contemporary European Studies. 2023;32(1):110–24. doi: 10.1080/14782804.2023.2198691

[60] Schwalbe CB. Visually framing the invasion and occupation of Iraq in Time, Newsweek, and US News & World Report. International Journal of Communication. 2013;7:239–62.

[61] Schwalbe CB, Dougherty SM. Visual coverage of the 2006 Lebanon War: Framing conflict in three US news magazines. Media, War & Conflict. 2015;8(1):141–62. doi: 10.1177/1750635215571204

[62] Urman A, Makhortykh M. My war is your special operation: Engagement with pro- and anti-regime framing of the war in Ukraine on Russian social media. OSF Preprints. 2022. doi: 10.31219/osf.io/67snk

[63] Kim J, Wanta W. News framing of the U.S. immigration debate during election years: Focus on generic frames. The Communication Review. 2018;21(2):89–115. doi: 10.1080/10714421.2018.1479616

[64] Houston JB, Pfefferbaum B, Rosenholtz CE. Disaster news: Framing and frame changing in coverage of major U.S. natural disasters, 2000–2010. Journalism & Mass Communication Quarterly. 2012;89(4):606–23. doi: 10.1177/1077699012456022

[65] Issac TF. A content analysis of the portrayal of refugees in U.S. news media. Provo (UT): Brigham Young University. 2017. https://scholarsarchive.byu.edu/etd/6621

[66] Makhortykh M, Urman A, Ulloa R. This Is What Pandemic Looks Like: Visual Framing of COVID-19 on Search Engines. COVID Communication. Springer International Publishing. 2023:113–23. doi: 10.1007/978-3-031-27665-1_9

[67] Wicke P, Bolognesi MM. Framing COVID-19: How we conceptualize and discuss the pandemic on Twitter. PLOS ONE. 2020;15(9):e0240010. doi: 10.1371/journal.pone.0240010 PMC7526906 32997720

[68] Semetko HA, Valkenburg PMV. Framing European politics: A Content Analysis of Press and Television News. Journal of Communication. 2000;50(2):93–109. doi: 10.1111/j.1460-2466.2000.tb02843.x

[69] Maas A. Media framing and tone of voice in Dutch newspapers: A comparative analysis of the Syrian and Ukrainian refugee crisis. Utrecht (NL): Utrecht University, Faculty of Social and Behavioral Sciences. 2024. https://studenttheses.uu.nl/bitstream/handle/20.500.12932/47233/maas_masterthesis23-24.pdf

[70] Abdelhady D. Framing the Syrian refugee: Divergent discourses in three national contexts. In: Menjívar C, Ruiz M, Ness I, editors. The Oxford Handbook of Migration Crises. Oxford (UK): Oxford University Press. 2019: 635–56. doi: 10.1093/oxfordhb/9780190856908.013.16

[71] Abdelhady D, Fristedt Malmberg G. Swedish media representation of the refugee crisis: Islam, conflict and self-reflection. In: O’Donnell E, Polyakov, editors. Anti-Judaism, Islamophobia, and interreligious hermeneutics: Ways of seeing the religious other. Leiden: Brill. 2018:107–36. doi: 10.1163/9789004381674_008

[72] Figenschou TU, Thorbjørnsrud K. Faces of an Invisible Population: Human Interest Framing of Irregular Immigration News in the United States, France, and Norway. American Behavioral Scientist. 2015;59(7):783–801. doi: 10.1177/0002764215573256

[73] Eberl J-M, Meltzer CE, Heidenreich T, Herrero B, Theorin N, Lind F, et al. The European media discourse on immigration and its effects: a literature review. Annals of the International Communication Association. 2018;42(3):207–23. doi: 10.1080/23808985.2018.1497452

[74] Abdullah G. Framing the Sudanese refugee flows to Egypt across traditional and social media. Cairo (EG): The American University in Cairo. 2025. https://fount.aucegypt.edu/etds/2448

[75] ALDayel A, Magdy W. Stance detection on social media: State of the art and trends. Information Processing & Management. 2021;58(4):102597. doi: 10.1016/j.ipm.2021.102597

[76] Küçük D, Can F. Stance Detection. ACM Comput Surv. 2020;53(1):1–37. doi: 10.1145/3369026

[77] Pivecka N, Ratzinger RA, Florack A. Emotions and virality: Social transmission of political messages on Twitter. Front Psychol. 2022;13:931921. doi: 10.3389/fpsyg.2022.931921 36438335 PMC9692101

[78] Zhang B, Dai G, Niu F, Yin N, Fan X, Wang S, et al. A survey of stance detection on social media: New directions and perspectives. 2024. doi: arXiv:2409.16256

[79] Hirsbrunner SD. Computational methods for climate change frame analysis: Techniques, critiques, and cautious ways forward. WIREs Climate Change. 2024;15(5). doi: 10.1002/wcc.902

[80] Zhao J, Tu J, Du H, Xue N. Media Attitude Detection via Framing Analysis with Events and their Relations. In: Proceedings of the 2024 Conference on Empirical Methods in Natural Language Processing, 2024. 17197–210. doi: 10.18653/v1/2024.emnlp-main.954

[81] Bazzaz Abkenar S, Haghi Kashani M, Mahdipour E, Jameii SM. Big data analytics meets social media: A systematic review of techniques, open issues, and future directions. Telemat Inform. 2021;57:101517. doi: 10.1016/j.tele.2020.101517 34887614 PMC7553883

[82] KhosraviNik M. Social media critical discourse studies (SM-CDS). In: Flowerdew C, Richardson J, editors. Handbook of Critical Discourse Analysis. London (UK): Routledge. 2017:582–96.

[83] López-Rabadán P. Framing Studies Evolution in the Social Media Era. Digital Advancement and Reorientation of the Research Agenda. Social Sciences. 2021;11(1):9. doi: 10.3390/socsci11010009

[84] Mendes K, Ringrose J, Keller J. #MeToo and the promise and pitfalls of challenging rape culture through digital feminist activism. European Journal of Women’s Studies. 2018;25(2):236–46. doi: 10.1177/1350506818765318

[85] Segerberg A, Bennett WL. Social Media and the Organization of Collective Action: Using Twitter to Explore the Ecologies of Two Climate Change Protests. The Communication Review. 2011;14(3):197–215. doi: 10.1080/10714421.2011.597250

[86] Shah Z, Surian D, Dyda A, Coiera E, Mandl KD, Dunn AG. Automatically Appraising the Credibility of Vaccine-Related Web Pages Shared on Social Media: A Twitter Surveillance Study. J Med Internet Res. 2019;21(11):e14007. doi: 10.2196/14007 31682571 PMC6862002

[87] Siapera E, Boudourides M, Lenis S, Suiter J. Refugees and Network Publics on Twitter: Networked Framing, Affect, and Capture. Social Media + Society. 2018;4(1). doi: 10.1177/2056305118764437

[88] Benford RD, Snow DA. Framing Processes and Social Movements: An Overview and Assessment. Annu Rev Sociol. 2000;26(1):611–39. doi: 10.1146/annurev.soc.26.1.611

[89] Druckman JN, Peterson E, Slothuus R. How Elite Partisan Polarization Affects Public Opinion Formation. Am Polit Sci Rev. 2013;107(1):57–79. doi: 10.1017/s0003055412000500

[90] Bonsignore EM, Dunne C, Rotman D, Smith M, Capone T, Hansen DL, et al. First Steps to Netviz Nirvana: Evaluating Social Network Analysis with NodeXL. In: 2009 International Conference on Computational Science and Engineering, 2009. 332–9. doi: 10.1109/cse.2009.120

[91] Hansen DL, Shneiderman B, Smith MA. Analyzing social media networks with NodeXL: Insights from a connected world. 2nd ed. Morgan Kaufmann. 2020.

[92] Smith MA, Shneiderman B, Milic-Frayling N, Mendes Rodrigues E, Barash V, Dunne C, et al. Analyzing (social media) networks with NodeXL. In: Proceedings of the fourth international conference on Communities and technologies, 2009. 255–64. doi: 10.1145/1556460.1556497

[93] Smith MA, Rainie L, Shneiderman B, Himelboim I. Mapping Twitter topic networks: From polarized crowds to community clusters. https://www.pewresearch.org/internet/2014/02/20/mapping-twitter-topic-networks-from-polarized-crowds-to-community-clusters/. 2014. Accessed 2024 October 21.

[94] Ahmed W, Lugovic S. Social media analytics: analysis and visualisation of news diffusion using NodeXL. OIR. 2019;43(1):149–60. doi: 10.1108/oir-03-2018-0093

[95] Maharani W, Adiwijaya, Gozali AA. Degree centrality and eigenvector centrality in twitter. In: 2014 8th International Conference on Telecommunication Systems Services and Applications (TSSA), 2014. 1–5. doi: 10.1109/tssa.2014.7065911

[96] Mitei E, Ghanem T. Leveraging Social Network Analysis to Explore Obesity Talks on Twitter. In: 2020 IEEE International Conference on Big Data (Big Data), 2020. 3563–72. doi: 10.1109/bigdata50022.2020.9377798

[97] Harel D, Koren Y. A Fast Multi-Scale Method for Drawing Large Graphs. Graph Algorithms and Applications 3. WORLD SCIENTIFIC. 2004:179–202. doi: 10.1142/9789812796608_0010

[98] White DR, Borgatti SP. Betweenness centrality measures for directed graphs. Social Networks. 1994;16(4):335–46. doi: 10.1016/0378-8733(94)90015-9

[99] Clauset A, Newman MEJ, Moore C. Finding community structure in very large networks. Phys Rev E Stat Nonlin Soft Matter Phys. 2004;70(6 Pt 2):066111. doi: 10.1103/PhysRevE.70.066111 15697438

[100] Olszowski R, Zabdyr-Jamróz M, Baran S, Pięta P, Ahmed W. A Social Network Analysis of Tweets Related to Mandatory COVID-19 Vaccination in Poland. Vaccines (Basel). 2022;10(5):750. doi: 10.3390/vaccines10050750 35632506 PMC9145409

[101] Boyd D, Golder S, Lotan G. Tweet, tweet, retweet: conversational aspects of retweeting on Twitter. In: 2010 43rd Hawaii International Conference on System Sciences. 2010. doi: 10.1109/HICSS.2010.412

[102] Park CS, Kaye BK. Expanding Visibility on Twitter: Author and Message Characteristics and Retweeting. Social Media + Society. 2019;5(2). doi: 10.1177/2056305119834595

[103] Sobhani P. Stance Detection and Analysis in Social Media. https://ruor.uottawa.ca/server/api/core/bitstreams/a035250f-4e15-4dd9-96c2-9cb6e108c635/content. 2017. Accessed 2024 September 14.

[104] Mohammad S, Kiritchenko S, Sobhani P, Zhu X, Cherry C. A dataset for detecting stance in tweets. In: Proceedings of the Tenth International Conference on Language Resources and Evaluation (LREC’16), Portorož, Slovenia, 2016. 3945–52. https://aclanthology.org/L16-1623

[105] Makhortykh M, Sydorova M. Social media and visual framing of the conflict in Eastern Ukraine. Media, War & Conflict. 2017;10(3):359–81. doi: 10.1177/1750635217702539

[106] Hameleers M. The visual nature of information warfare: the construction of partisan claims on truth and evidence in the context of wars in Ukraine and Israel/Palestine. Journal of Communication. 2024;75(2):90–100. doi: 10.1093/joc/jqae045

[107] Ward JH. Hierarchical grouping to optimize an objective function. Journal of the American Statistical Association. 1963;58(301):236–44. doi: 10.1080/01621459.1963.10500845

[108] Ferrara E, Yang Z. Measuring Emotional Contagion in Social Media. PLoS One. 2015;10(11):e0142390. doi: 10.1371/journal.pone.0142390 26544688 PMC4636231

[109] Stieglitz S, Dang-Xuan L. Emotions and Information Diffusion in Social Media—Sentiment of Microblogs and Sharing Behavior. Journal of Management Information Systems. 2013;29(4):217–48. doi: 10.2753/mis0742-1222290408

[110] Case CJ, King DL. Internet trolling victimization: An empirical examination of incidence in undergraduate business students. Research in Higher Education Journal. 2018;34:1–11.

[111] Scriven P. Online trolling as a dark leisure activity. Annals of Leisure Research. 2024;:1–19. doi: 10.1080/11745398.2024.2358764

[112] Nasuto A, Rowe F. Understanding anti-immigration sentiment spreading on Twitter. PLoS One. 2024;19(9):e0307917. doi: 10.1371/journal.pone.0307917 39231099 PMC11373840

[113] Moise AD, Dennison J, Kriesi H. European attitudes to refugees after the Russian invasion of Ukraine. West European Politics. 2023;47(2):356–81. doi: 10.1080/01402382.2023.2229688

[114] De Coninck D. The Refugee Paradox During Wartime in Europe: How Ukrainian and Afghan Refugees are (not) Alike. International Migration Review. 2022;57(2):578–86. doi: 10.1177/01979183221116874

[115] Sikt - Norwegian Agency for Shared Services in Education and Research. European Social Survey Round 10 Data. Data Archive and Distributor of ESS Data for ESS ERIC. Norway: Sikt - Norwegian Agency for Shared Services in Education and Research. 2020. doi: 10.21338/NSD-ESS10-2020

[116] Weber M, Grunow D, Chen Y, Eger S. Social solidarity with Ukrainian and Syrian refugees in the twitter discourse. A comparison between 2015 and 2022. European Societies. 2023;26(2):346–73. doi: 10.1080/14616696.2023.2275604

[117] Thiele D, Turnšek T. How Right-Wing Populist Comments Affect Online Deliberation on News Media Facebook Pages. MaC. 2022;10(4):141–54. doi: 10.17645/mac.v10i4.5690

[118] Rush N. Ukrainian refugees are not like the others: An overwhelmingly female flow presents unique challenges for integration. https://cis.org/Oped/Ukrainian-Refugees-Are-Not-Others. 2023. Accessed 2024 August 2.

[119] Fiske ST, Cuddy AJC, Glick P, Xu J. A model of (often mixed) stereotype content: competence and warmth respectively follow from perceived status and competition. J Pers Soc Psychol. 2002;82(6):878–902. doi: 10.1037/0022-3514.82.6.878 12051578

[120] Lee TL, Fiske ST. Not an outgroup, not yet an ingroup: Immigrants in the Stereotype Content Model. International Journal of Intercultural Relations. 2006;30(6):751–68. doi: 10.1016/j.ijintrel.2006.06.005

[121] Davis N. White eagle, red star: The Polish–Soviet war, 1919–20. London: Macdonald & Co. 1972.

[122] Fagan A, Kopecký P. The Routledge Handbook of East European Politics. Routledge. 2017. doi: 10.4324/9781315687681

[123] Golovashina OV. Battles for Bandera: Dissonant Historical Narratives of Ukrainians in Poland and Problems of Integration. ChS&P. 2021;5(3):355–71. doi: 10.15826/csp.2021.5.3.139

[124] Kruk MS. Economic immigration in the secondary segment in Poland taking the example of employees from Ukraine. Migration Letters. 2022;19(6). doi: 10.33182/ml.v19i6.1404

[125] Lehmann R. From ethnic cleansing to affirmative action: exploring Poland’s struggle with its Ukrainian minority (1944-89). Nations and Nationalism. 2010;16(2):285–307. doi: 10.1111/j.1469-8129.2010.00439.x

[126] Snyder T. “To Resolve the Ukrainian Problem Once and for All”: The Ethnic Cleansing of Ukrainians in Poland, 1943–1947. Journal of Cold War Studies. 1999;1(2):86–120. doi: 10.1162/15203979952559531

[127] Snyder T. The Reconstruction of Nations: Poland, Ukraine, Lithuania, Belarus, 1569–1999. New Haven (CT): Yale University Press. 2003.

[128] Strzelecki P, Growiec J, Wyszyński R. The contribution of immigration from Ukraine to economic growth in Poland. Rev World Econ. 2021;158(2):365–99. doi: 10.1007/s10290-021-00437-y

[129] Waingertner P. The Eastern European Order in the Polish Political Thought of the 20th Century. Berlin: Peter Lang. 2021. doi: 10.3726/b17723

