# Supplementary material for: Framing the crisis: X/Twitter discourse on Ukrainian war refugees in Poland
Source: PLoS One. 2026 May 5;21(5):e0346666. doi: 10.1371/journal.pone.0346666 (PMC13143064; doi:10.1371/journal.pone.0346666)
Supplement: S1 Text — (DOCX) [file pone.0346666.s001.docx]

S1 Text

**BRIEF BACKGROUND ON POLISH-UKRAINIAN RELATIONS**

Since the disintegration of the Union of Soviet Socialist Republics (USSR), safeguarding Ukraine’s independence has been widely acknowledged by Polish scholars and policy-makers as a strategic priority in foreign policy, in line with the so-called *Giedroyc Doctrine*, which emphasized the necessity of an independent and sovereign Ukraine as a cornerstone of Poland's long-term security and regional stability [39, 119] . All Polish governments over the past 30 years have supported Ukraine’s integration into Western international structures, recognizing the possibility of its accession to the European Union and the North Atlantic Treaty Organization (NATO). Particularly over the past decade, both countries have been united by a shared awareness of the potential threats posed by Russia.

The western territories of present-day Ukraine were part of the Polish state for many centuries and were home to a diverse, multi-ethnic society. The relationships between the nations inhabiting these lands were complex, oscillating between periods of peaceful co-existence and tensions driven by religious and ethnic differences. In 1918, Poles defended the area against an attack by Ukrainian forces, which sought to establish their own state after the collapse of the Habsburg Empire. In 1919–1920, Poland and Ukraine engaged in a short-lived military alliance to repel Soviet revolutionary forces from invading the territory of the newly formed Ukrainian state [23]. This entente ended with the defeat of Ukrainian independence troops and Poland’s decision to break off military co-operation, making peace with communist Russia in the second half of 1920 to secure its own independence. Before World War II, Ukrainians made up about 16% of Poland's population and were an integral part of the cultural landscape of a diverse society [106].

During World War II, some Ukrainians collaborated with the German occupation authorities in an attempt to regain their independence and turned against the Polish population residing in what is now western Ukraine. These actions culminated in the Slaughter of Volhynia, which involved the forced displacement of the Polish population and large-scale killings [46, 106]. After the war, primarily as a result of border shifts, Poland became a largely mono-ethnic state. Between 1944 and 1947, the Soviet-controlled Polish government forcibly deported around 482,000 Ukrainians to the USSR [105]. Until the fall of the Iron Curtain in 1989, Ukrainian migration to Poland remained rare due to the heavily restricted Soviet border [67].

The situation changed following Ukraine's declaration of independence in 1991. At the turn of the century, Poland, as a more developed country increasingly integrated into Western structures, began to attract immigrants from Ukraine; however, this process progressed gradually [64, 110]. In 2002, 96.7% of the Polish population identified as Polish by nationality, a figure that declined to 89.2% by 2021 [108].
