# Supplementary material for: Framing the crisis: X/Twitter discourse on Ukrainian war refugees in Poland
Source: PLoS One. 2026 May 5;21(5):e0346666. doi: 10.1371/journal.pone.0346666 (PMC13143064; doi:10.1371/journal.pone.0346666)
Supplement: S1 Table — (DOCX) [file pone.0346666.s002.docx]

S1 Table

**THE CRITERIA FOR IDENTIFYING ISSUE-SPECIFIC FRAMES**

| Issue-specific frame | Question used to identify the frame | Follow-up questions |
| --- | --- | --- |
| Migration flows | Does the story concern the influx or outflow of Ukrainians? | Does it refer to the number, direction or intensity of refugee movements? Does it describe border crossings, arrivals, departures or returns? |
| Migrant features | Does the story highlight the characteristics of migrants? | Does it mention their behaviour, appearance, personal culture, diligence, gratitude or lack thereof? Does it refer to stereotypes? |
| Helping Ukrainians, bottom up (society) and top down  (internal policy) | Does the story discuss grassroots and governmental support? | Does it mention actions taken activities of the government, local authorities, NGOs or citizens in helping Ukrainians (housing, benefits, work, education)? |
| Second World War | Does the story relate to the Second World War? | Does it refer to the history of Volhynia? Does it link historical events with the current situation? |
| International relations | Does the story emphasise the impact of the issue/problem on international relations? | Does it refer to Poland's relations with Ukraine, the US, the EU, NATO, and Russia? 2. Does it concern foreign policy and the geopolitical position of countries? Does it mention meetings between political leaders and military aid? |
